# A Case of New-Onset Atrial Tachyarrhythmias With Apical Hypertrophic Cardiomyopathy and Bronchiectasis in a Very Elderly Patient: A Therapeutic Dilemma

**DOI:** 10.7759/cureus.63272

**Published:** 2024-06-27

**Authors:** Satoshi Kurisu, Hitoshi Fujiwara

**Affiliations:** 1 Department of Cardiology, National Hospital Organization (NHO) Hiroshima-Nishi Medical Center, Otake, JPN

**Keywords:** case report, hemoptysis, anticoagulation, arrhythmia, aging

## Abstract

Hypertrophic cardiomyopathy (HCM) is a primary myocardial disease that is genetically transmitted as an autosomal dominant trait. Even apical HCM (ApHCM) induces atrial fibrillation (AF) based on underlying left ventricle (LV) diastolic dysfunction, where anticoagulation therapy is recommended. However, anticoagulation for AF in patients at high risk of bleeding is a double-edged sword. A 98-year-old woman living in a nursing home presented to our hospital with sudden-onset dyspnea and palpitation persisting for two hours. The patient had a history of apical HCM and bronchiectasis. An electrocardiogram showed a regular tachycardia with a heart rate of 130 bpm, suggesting atrial flutter with 2:1 atrioventricular conduction. Intravenous verapamil (5 mg) resulted in the conversion into AF, and subsequent cibenzoline (70 mg) failed to restore sinus rhythm. Given the impossibility of continuous anticoagulation, electrical cardioversion was planned. Electrical cardioversion was successful in converting AF into sinus rhythm. Given the very high risk of hemoptysis, anticoagulation was avoided. This case gives an insight into how to manage a practical therapeutic problem, which is the coexistence of AF and bronchiectasis. A variety of individual factors should be considered for clinical decision-making and management of patients with concomitant HCM and AF.

## Introduction

Hypertrophic cardiomyopathy (HCM) is a primary myocardial disease that is genetically transmitted as an autosomal dominant trait [1,2]. Clinical course is variable, ranging from asymptomatic disease, to heart failure, stroke, and sudden cardiac death [1-5]. Apical HCM (ApHCM) is a common type of HCM in Japan, in which the hypertrophy predominantly involves the apex of the left ventricle (LV) [1,2]. A previous study using cardiac magnetic resonance imaging revealed that ApHCM had a relatively small burden of myocardial fibrosis and less LV diastolic dysfunction [4]. This may be one reason why most patients with ApHCM show a benign course of disease compared to those with non-ApHCM [4]. However, even ApHCM induces atrial fibrillation (AF) based on underlying LV diastolic dysfunction [6,7]. Anticoagulation, which reduces thromboembolic events compared to no treatment in patients with concomitant HCM and AF, is recommended [8].

Bronchiectasis is a chronic, progressive lung disease due to a vicious cycle of infection and inflammation, leading to bronchial dilatation and wall thickening [9,10]. Bronchiectasis is one of the most common causes of massive hemoptysis, which is a life-threatening condition with high mortality rates [11].

Anticoagulation for AF in patients at high risk of bleeding is a double-edged sword. It requires careful decision-making and management with multidisciplinary expertise. Herein, we reported a case of new-onset atrial tachyarrhythmias with known ApHCM and bronchiectasis in a very elderly patient, which posed a therapeutic dilemma.

## Case presentation

A-98-year-old woman living in a nursing home presented to our hospital with sudden-onset dyspnea and palpitation persisting for two hours. The patient had a history of ApHCM and bronchiectasis.

**Figure 1:**
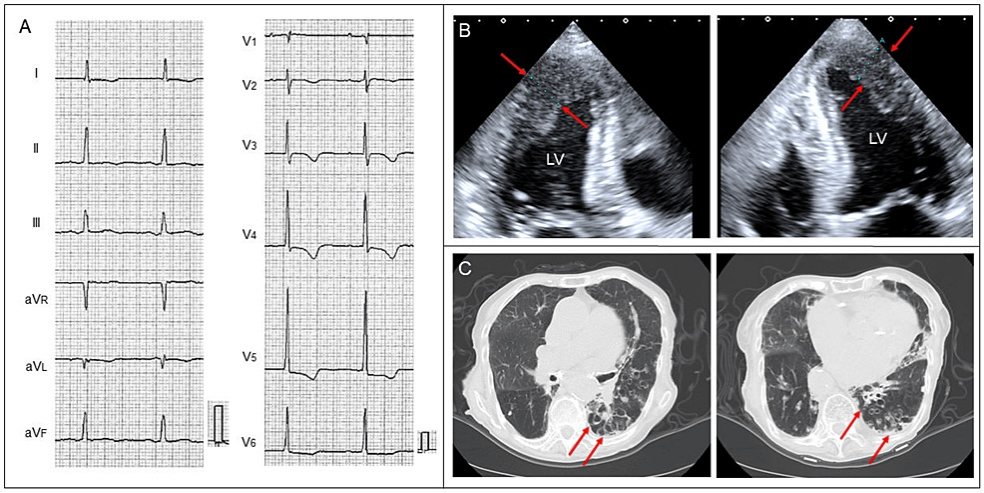
Previously obtained images A diagnosis of apical hypertrophic cardiomyopathy had been made two years before based on electrocardiographic and echocardiographic findings such as high voltage, inverted T-wave (A), and apical wall thickness of 15 mm (B, arrows). A diagnosis of bronchiectasis had been made 10 years before based on computed tomographic findings such as bronchial dilatation and nodular shadow predominantly in the left lower lobe (C, arrows). LV, left ventricle

A diagnosis of ApHCM had been made two years before based on electrocardiographic (ECG) and echocardiographic findings such as high voltage, inverted T-wave (Figure 1A), and apical wall thickness of 15 mm (Figure 1B). Left atrial enlargement with a volume index of 86 mL/m2 had been identified at that time. A diagnosis of bronchiectasis had been made 10 years before based on computed tomographic findings such as bronchial dilatation and nodular shadow predominantly in the left lower lobe (Figure 1C). Bronchiectasis-related hemoptysis had been followed up conservatively.

**Figure 2:**
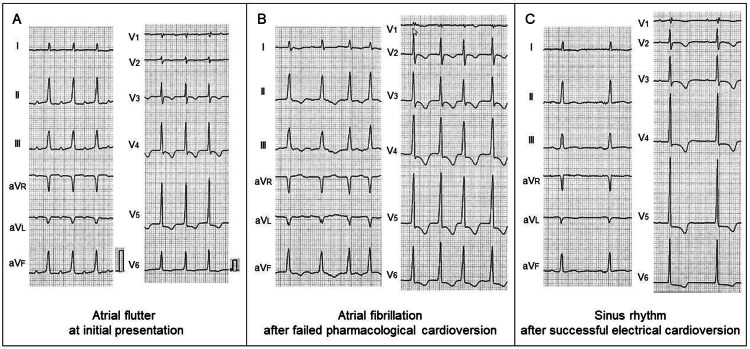
Serial electrocardiograms Serial electrocardiograms at initial presentation (A), after failed pharmacological cardioversion (B), and after successful electrical cardioversion (C).

At initial presentation, her pulse rate was 130 bpm and blood pressure was 90/64 mmHg. Oxygen saturation was 94%. She did not have peripheral edema. Her body weight was 34 kg, and body mass index was 17.8 kg/m^2^. Laboratory studies showed the following values: white blood cell count of 7,500/μL, hemoglobin of 11.7 g/dL, creatinine of 0.59 mg/dL, aspartate aminotransferase of 20 U/L, alanine aminotransferase of 9 U/L, creatine phosphokinase of 73 U/L, and C-reactive protein of 0.20 mg/dL. There were no significant abnormal values. An ECG showed a regular tachycardia with a heart rate of 130 bpm, suggesting new-onset atrial flutter with 2:1 atrioventricular conduction (Figure 2A). A transthoracic echocardiogram showed no wall motion abnormalities. There were no echocardiographic findings suggestive of intracardiac thrombus. Intravenous verapamil (5 mg) resulted in the conversion into AF, and subsequent cibenzoline (70 mg) failed to restore sinus rhythm (Figure 2B). She was admitted for careful follow-up. Five hours after the onset of palpitation, AF still persisted with rapid ventricular response. Given the impossibility of continuous anticoagulation, electrical cardioversion was planned. Considering the patient’s age, frailty, and time from onset, evaluation by transesophageal echocardiography was skipped. Synchronized direct current cardioversion was successful in converting AF into sinus rhythm (Figure 2C). No adverse cardiac events occurred.

**Figure 3:**
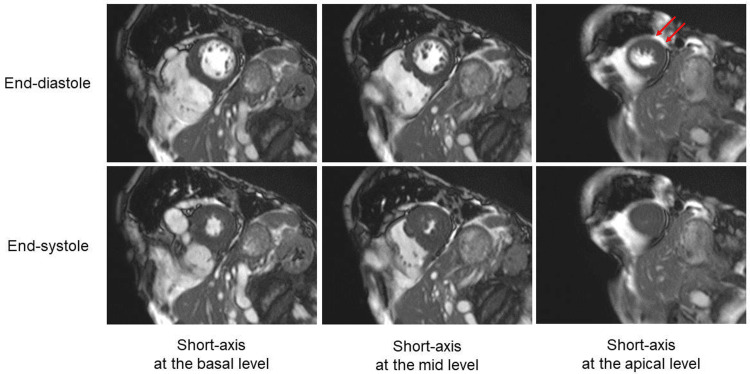
Cardiac magnetic resonance imaging Cardiac magnetic resonance imaging showed asymmetric LV hypertrophy confined predominantly to the apex, with an apical wall thickness of 15 mm (arrows) and a ratio of maximal apical to posterior wall thickness of 1.7. LV, left ventricle

Subsequently, the patient was treated with oral verapamil (80 mg/day) and cibenzoline (100 mg/day) to maintain sinus rhythm. Given the very high risk of hemoptysis, anticoagulation was avoided. Three days after the cardioversion, cardiac magnetic resonance imaging was performed, showing asymmetric LV hypertrophy confined predominantly to the apex, with an apical wall thickness of 15 mm and a ratio of maximal apical to posterior wall thickness of 1.7 (Figure 3). Based on these findings, a definite diagnosis of ApHCM was made.

The patient returned to a nursing home and remained in good condition without the recurrence of atrial tachyarrhythmias.

## Discussion

In this report, we showed the case of new-onset atrial tachyarrhythmias with known ApHCM and bronchiectasis in a very elderly patient. Electrical cardioversion was successful in converting into sinus rhythm and contributed to the avoidance of subsequent anticoagulation.

A unique aspect of this case was that the patient had never experienced cardiac events until the age of 98 years in spite of the presence of ApHCM. According to a previous report focused on the long-term outcome of patients with ApHCM [3], the most common morbid event was AF, and left atrial enlargement was the only predictor of AF. Lee et al. reported that AF occurred in 25.2% of the patients with ApHCM (annual incidence 4.6%/years) and was predicted by old age and left atrial enlargement [6]. Our patient’s clinical course was consistent with the existing knowledge regarding ApHCM. According to a recent study focused on AF in the general HCM population [7], 29% of the patients at registration had documentation of AF, and the annual detection rate of new-onset AF was 1.8% during the follow-up period. The presence of AF, particularly new-onset AF, was associated with unfavorable clinical outcomes such as heart failure or thromboembolism. In the present case, such documented risk of AF contributed to aggressive electrical cardioversion in spite of the advanced age of 98 years. Consequently, successful electrical cardioversion resulted in the avoidance of anticoagulation, contributing to reducing the risk of bleeding.

The other unique aspect of this case was the coexistence of AF and bronchiectasis. It posed a therapeutic dilemma for our patient. Guttmann et al. previously reported that CHA2DS2-VASc (congestive heart failure, hypertension, age ≥75years, diabetes mellitus, prior stroke or TIA or thromboembolism, vascular disease, age between 65-74 years, sex category) score, which is recommended for stratifying patients with AF for antithrombotic prophylaxis, did not correlate with embolic events in patients with HCM [12]. Hirota et al. also recently reported that CHADS2 score had a low predictive accuracy in Japanese patients with HCM [13]. These results suggest that HCM itself is a major risk factor associated with thromboembolism, independent of factors involved in CHA2DS2-VASc or CHADS2 score. On the other hand, anticoagulation is usually avoided in case of hemoptysis because it increases the chances of bleeding. A previous study showed that 27% of patients with bronchiectasis developed hemoptysis [14]. A near-fatal case of bronchiectasis with massive hemoptysis following anticoagulation was also reported [15]. Anticoagulation is a double-edged sword especially in patients at high risk of bleeding. In the present case, the patient had two factors of “bleeding history of predisposition” and “elderly” involved in the HAS-BLED (hypertension, abnormal renal/liver function, stroke, bleeding history or predisposition, labile international normalized ratio [INR], elderly [age ≥65 years], drugs/alcohol concomitantly) bleeding risk score system. Given the very high risk for bleeding, subsequent anticoagulation was avoided.

 As for antiarrhythmic drugs in patients with HCM, amiodarone has been shown to be effective in maintaining sinus rhythm [16,17]. Class I antiarrhythmic drugs such as disopyramide or cibenzoline have been used to reduce the pressure gradient in LV outflow tract obstruction, whereas their effects on maintaining sinus rhythm are unclear [1]. In the present case, the use of amiodarone was avoided because pulmonary toxicity, a serious side-effect of amiodarone, could be fatal under the condition of bronchiectasis [18]. Verapamil and cibenzoline were initiated after successful cardioversion in expectation of maintaining sinus rhythm and further improving LV diastolic dysfunction [19,20].

Because AF is a common condition in patients with HCM, clinicians should take care of this arrhythmia during regular follow-up. The mainstay of management of AF in HCM is a combination of lifestyle modification, anticoagulation, rhythm control with antiarrhythmic drugs, and catheter ablation [21]. Considering each patient’s background such as age and risk of bleeding, clinicians should provide appropriate management on a case-by-case basis.

## Conclusions

In conclusion, we reported the case of new-onset atrial tachyarrhythmias with known ApHCM and bronchiectasis in a very elderly patient. Electrical cardioversion was successful in converting into sinus rhythm and contributed to the avoidance of subsequent anticoagulation. This case gives an insight into how to manage a practical therapeutic problem, which is the coexistence of AF and bronchiectasis. A variety of individual factors should be considered for clinical decision-making and management of patients with concomitant HCM and AF.

## References

[1] Kitaoka H, Tsutsui H, Kubo T (2021). JCS/JHFS 2018 Guideline on the Diagnosis and Treatment of Cardiomyopathies. Circ J.

[2] Kubo T, Hirota T, Baba Y (2018). Patients' Characteristics and clinical course of hypertrophic cardiomyopathy in a regional Japanese cohort - results from Kochi RYOMA study. Circ J.

[3] Eriksson MJ, Sonnenberg B, Woo A, Rakowski P, Parker TG, Wigle ED, Rakowski H (2002). Long-term outcome in patients with apical hypertrophic cardiomyopathy. J Am Coll Cardiol.

[4] Kim EK, Lee SC, Hwang JW (2016). Differences in apical and non-apical types of hypertrophic cardiomyopathy: a prospective analysis of clinical, echocardiographic, and cardiac magnetic resonance findings and outcome from 350 patients. Eur Heart J Cardiovasc Imaging.

[5] Rouskas P, Zegkos T, Ntelios D (2023). Prevalence, characteristics, and natural history of apical phenotype in a large cohort of patients with hypertrophic cardiomyopathy. Hellenic J Cardiol.

[6] Lee SE, Park JK, Uhm JS, Kim JY, Pak HN, Lee MH, Joung B (2017). Impact of atrial fibrillation on the clinical course of apical hypertrophic cardiomyopathy. Heart.

[7] Kubo T, Baba Y, Ochi Y (2021). Clinical significance of new-onset atrial fibrillation in patients with hypertrophic cardiomyopathy. ESC Heart Fail.

[8] Lozier MR, Sanchez AM, Lee JJ, Donath EM, Font VE, Escolar E (2019). Thromboembolic outcomes of different anticoagulation strategies for patients with atrial fibrillation in the setting of hypertrophic cardiomyopathy: a systematic review. J Atr Fibrillation.

[9] Chalmers JD, Smith MP, McHugh BJ, Doherty C, Govan JR, Hill AT (2012). Short- and long-term antibiotic treatment reduces airway and systemic inflammation in non-cystic fibrosis bronchiectasis. Am J Respir Crit Care Med.

[10] Aksamit TR, O'Donnell AE, Barker A (2017). Adult patients with bronchiectasis: a first look at the US Bronchiectasis Research Registry. Chest.

[11] Luo L, Luo J, Jiang Y (2022). A retrospective analysis of risk factors for massive hemoptysis in patients with bronchiectasis. BMC Pulm Med.

[12] Guttmann OP, Pavlou M, O'Mahony C (2015). Prediction of thrombo-embolic risk in patients with hypertrophic cardiomyopathy (HCM Risk-CVA). Eur J Heart Fail.

[13] Hirota T, Kubo T, Baba Y (2019). Clinical profile of thromboembolic events in patients with hypertrophic cardiomyopathy in a regional Japanese cohort - results from Kochi RYOMA study. Circ J.

[14] King PT, Holdsworth SR, Freezer NJ, Villanueva E, Holmes PW (2006). Characterisation of the onset and presenting clinical features of adult bronchiectasis. Respir Med.

[15] Hayama M, Inoue H, Wada H, Mio T (2014). Massive haemoptysis following dabigatran administration in a patient with bronchiectasis. BMJ Case Rep.

[16] McKenna WJ, Harris L, Rowland E, Kleinebenne A, Krikler DM, Oakley CM, Goodwin JF (1984). Amiodarone for long-term management of patients with hypertrophic cardiomyopathy. Am J Cardiol.

[17] Robinson K, Frenneaux MP, Stockins B, Karatasakis G, Poloniecki JD, McKenna WJ (1990). Atrial fibrillation in hypertrophic cardiomyopathy: a longitudinal study. J Am Coll Cardiol.

[18] Ott MC, Khoor A, Leventhal JP, Paterick TE, Burger CD (2003). Pulmonary toxicity in patients receiving low-dose amiodarone. Chest.

[19] Matsubara H, Nakatani S, Nagata S, Ishikura F, Katagiri Y, Ohe T, Miyatake K (1995). Salutary effect of disopyramide on left ventricular diastolic function in hypertrophic obstructive cardiomyopathy. J Am Coll Cardiol.

[20] Hamada M, Shigematsu Y, Ikeda S (1997). Class Ia antiarrhythmic drug cibenzoline: a new approach to the medical treatment of hypertrophic obstructive cardiomyopathy. Circulation.

[21] Palyam V, Azam AT, Odeyinka O, Alhashimi R, Thoota S, Ashok T, Sange I (2022). Hypertrophic cardiomyopathy and atrial fibrillation: a review. Cureus.

